# Patient-Specific Computational Analysis of Hemodynamics and Wall Mechanics and Their Interactions in Pulmonary Arterial Hypertension

**DOI:** 10.3389/fbioe.2020.611149

**Published:** 2021-01-28

**Authors:** Byron A. Zambrano, Nathan McLean, Xiaodan Zhao, Ju-Le Tan, Liang Zhong, C. Alberto Figueroa, Lik Chuan Lee, Seungik Baek

**Affiliations:** ^1^J. Mike Walker '66 Department of Mechanical Engineering, Texas A&M University, College Station, TX, United States; ^2^Department of Mechanical Engineering, Michigan State University, East Lansing, MI, United States; ^3^National Heart Centre Singapore, Singapore, Singapore; ^4^Duke-National University of Singapore, Singapore, Singapore; ^5^Departments of Biomedical Engineering and Surgery, University of Michigan, Ann Arbor, MI, United States

**Keywords:** pulmonary arterial hypertension, fluid structure interaction, hemodynamics, pulmonary stiffness, biomechanics metrics

## Abstract

Vascular wall stiffness and hemodynamic parameters are potential biomechanical markers for detecting pulmonary arterial hypertension (PAH). Previous computational analyses, however, have not considered the interaction between blood flow and wall deformation. Here, we applied an established computational framework that utilizes patient-specific measurements of hemodynamics and wall deformation to analyze the coupled fluid–vessel wall interaction in the proximal pulmonary arteries (PA) of six PAH patients and five control subjects. Specifically, we quantified the linearized stiffness (*E*), relative area change (RAC), diastolic diameter (*D*), regurgitant flow, and time-averaged wall shear stress (TAWSS) of the proximal PA, as well as the total arterial resistance (*R*_*t*_) and compliance (*C*_*t*_) at the distal pulmonary vasculature. Results found that the average proximal PA was stiffer [median: 297 kPa, interquartile range (IQR): 202 kPa vs. median: 75 kPa, IQR: 5 kPa; *P* = 0.007] with a larger diameter (median: 32 mm, IQR: 5.25 mm vs. median: 25 mm, IQR: 2 mm; *P* = 0.015) and a reduced RAC (median: 0.22, IQR: 0.10 vs. median: 0.42, IQR: 0.04; *P* = 0.004) in PAH compared to our control group. Also, higher total resistance (*R*_*t*_; median: 6.89 mmHg × min/l, IQR: 2.16 mmHg × min/l vs. median: 3.99 mmHg × min/l, IQR: 1.15 mmHg × min/l; *P* = 0.002) and lower total compliance (*C*_*t*_; median: 0.13 ml/mmHg, IQR: 0.15 ml/mmHg vs. median: 0.85 ml/mmHg, IQR: 0.51 ml/mmHg; *P* = 0.041) were observed in the PAH group. Furthermore, lower TAWSS values were seen at the main PA arteries (MPAs) of PAH patients (median: 0.81 Pa, IQR: 0.47 Pa vs. median: 1.56 Pa, IQR: 0.89 Pa; *P* = 0.026) compared to controls. Correlation analysis within the PAH group found that *E* was directly correlated to the PA regurgitant flow (*r* = 0.84, *P* = 0.018) and inversely related to TAWSS (*r* = −0.72, *P* = 0.051). Results suggest that the estimated elastic modulus *E* may be closely related to PAH hemodynamic changes in pulmonary arteries.

## Introduction

Pulmonary arterial hypertension (PAH) is a complex cardiovascular disease characterized by a progressive remodeling of the pulmonary arteries. This ongoing process, which is promoted by an increase in pulmonary arterial (PA) pressure, leads to right atrial dysfunction (Leng et al., 2019), right ventricular (RV) hypertrophy (Driessen et al., 2018), and impaired ventricular–vascular coupling (Zhao et al., 2019). Although the end stage of PAH is right heart failure (van der Bruggen et al., 2017), this is driven mainly by pathogenesis of the pulmonary vasculature. Due to the highly complex environment and the ability of the cardiopulmonary system to adapt to adverse conditions, the onset symptoms are usually mild and may be difficult to identify (Humbert et al., 2012). As a consequence, PAH patients are commonly diagnosed at advanced stages, with a faster deterioration (3–5 years from diagnosed time to failure) (Thenappan et al., 2012) and higher mortality rates (29% within 5 years; Hoeper et al., 2017). Clinical guidance suggests that an early detection can greatly increase the chances of survival (Humbert et al., 2012; Vachiéry et al., 2012; Sundnes et al., 2014). As such, there is a pressing need to further understand the mechanisms leading to the progression of disease that can help identifying new biomechanical markers of PAH detection, especially at the earlier stages.

Recent evidence has suggested that vascular stiffening, right atrium (RA) and RV functions, and hemodynamics in the PA can serve as potential biomechanical markers for detection of PAH and prediction of its progression (Sanz et al., 2009; Swift et al., 2012, 2013; Bertero et al., 2015; Sun and Chan, 2018; Leng et al., 2019). In terms of vascular stiffening, parameters such as pulse wave velocity (Sanz et al., 2008; Kopeć et al., 2013; Prins et al., 2016), function (Gupta et al., 2018), relative area change (RAC) (Swift et al., 2012, 2017; Tian et al., 2014), stiffness index (Gan et al., 2007; Sanz et al., 2009; Stevens et al., 2012), and PA distensibility (Hayoz et al., 1992; Hayashi and Naiki, 2009; Ray et al., 2019) have been proposed. These indices, however, are not able to distinguish the influence of geometrical and material changes associated with vascular stiffening, i.e., they cannot assess changes in the intrinsic mechanical property of the vascular wall, which are suggested to be one of the main drivers of the progression of PAH (Sun and Chan, 2018).

In terms of hemodynamics and RV function, changes in indices such as RV end-diastolic volume (Amsallem et al., 2017), ejection fraction (Sandoval et al., 1994; van Wolferen et al., 2007; Champion et al., 2009; Swift et al., 2017), tricuspid annular motion (Leng et al., 2016), pulmonary vascular resistance (PVR), mean PA, and pulmonary capillary wedge pressures (Saggar and Sitbon, 2012) are commonly used to assess the progression of PAH. While RV end-diastolic volume can be measured non-invasively, its changes may become prominent only at advanced stages of the disease. The use of PA hemodynamic metrics, on the other hand, requires invasive right heart catheterization (RHC). The ability of these markers to serve as biomechanical metrics to identify PAH may therefore be limited.

Other non-invasive PA hemodynamic measurements have been proposed, e.g., changes in volumetric flow waveform profiles (Finley and van Doesburg, 1993). Specifically, reverse flow patterns found in the PA of PAH have been suggested as a potential biomarker given that it can be identified prior to other PAH indicators (Jones et al., 2002). Also, low wall shear stress (WSS) at the proximal PA is suggested as a good indicator of PAH severity (Smith et al., 2013) since it is shown to play a role in the pathogenesis of PAH (Tang et al., 2012; Truong et al., 2013). Some of these suggestions, however, have been made based on computational analyses that assume the compliant PA wall to be rigid (Tang et al., 2012; Smith et al., 2013), which is a limitation that has been previously discussed (Bordones et al., 2018; Kong et al., 2019).

Other computational models describing a patient-specific vessel wall–hemodynamic interaction have been recently used to study PAH in a single adult (Kong et al., 2019) or a pediatric cohort (Yang et al., 2019) of patients. However, there were only few studies investigating the hemodynamic–wall mechanics relationship in a cohort of adult patients. Hence, the objective of this study is to elicit the vascular wall mechanics and hemodynamic interaction at the proximal PA arteries of six PAH patients and five control subjects by exploiting a patient-specific fluid–structure interaction (FSI) framework that was previously developed by our group (Zambrano et al., 2018). The framework originally applied to assess the local hemodynamic–wall mechanics interaction in a PAH patient and a control subject (Zambrano et al., 2018) is used to quantify the linearized elastic modulus, wall surface displacements, hemodynamics, as well as the distal vascular compliance and resistance in multiple adult PAH patients.

## Methods

### Patient Cohort and Data

Cine and phase contrast (PC) magnetic resonance (MR) images were acquired using a 3-Tesla Philips scanner with ECG gating from six patients diagnosed with PAH based on catheterization. Blood velocities and volumetric flow rates at the main pulmonary artery (MPA) were post-processed from the PC-MR images using Q-Flow software (Philips Medical Systems). Right heart catheterization (RHC) was also performed at rest (within 1 week of imaging examination) on the PAH patients using standard techniques. Five human subjects with no known cardiovascular disease or other co-morbidities served as control in this study and also underwent MR imaging from which cine MR images were acquired. Because invasive hemodynamics measurements and PC-MR images were not acquired in the control group, the MPA pressure and volumetric flow waveforms measured in healthy human subjects from a previous study was used (Lankhaar et al., 2006). However, this characteristic volumetric flow waveform was adjusted to match the total volume outflow corresponding to each patient RV stroke volume measured from cine MR images. All data were acquired at the National Heart Center of Singapore, and demographics of the two study groups (PAH vs. control) are summarized in Table 1. The protocol was approved by the SingHealth Centralised Institutional Review Board, and informed consents were obtained from all subjects.

**Table 1:** Demographics and clinical examinations in PAH and normal control subjects^**^.

**Variables**	**Control (*n* = 5)**	**PAH (*n* = 6)**	***P* value**
**Demographics**			
Age, years	51 ± 15	50 ± 11	0.792
Gender, male/female	1/5	1/4	0.727
Weight, kg	66 ± 18	61 ± 10	0.662
Height, cm	160 ± 8	162 ± 6	0.792
**Clinical Exam**			
Body surface area, m^2^	1.70 ± 0.26	1.65 ± 0.12	0.931
Body mass index, kg/m^2^	25.2 ± 4.3	23.2 ± 4.7	0.177
6-min walking test, m	–	391 ± 103	–
NYHA functional class I, *n*	–	1	–
NYHA functional class II, *n*	–	4	–
NYHA functional class III, *n*	–	1	–
NYHA functional class IIII, *n*	–	0	–
**Cardiac magnetic resonance**			
LV ejection fraction, %	64 ± 6	60 ± 8	0.329
LVEDV, ml	111 ± 15	113 ± 18	0.931
LVESV, ml	40 ± 8	46 ± 14	0.792
LVSV, ml	71 ± 11	67 ± 12	0.537
RV ejection fraction, %	60 ± 9	45 ± 17	0.177
RVEDV, ml	118 ± 24	189 ± 114	0.537
RVESV, ml	49 ± 18	119 ± 104	0.329
RVSV, ml	69 ± 12	71 ± 16	0.931
**Hemodynamics**			
Heart rate, bpm	81 ± 17	71 ± 16	1
Diastolic blood pressure, mmHg	80 ± 17	74 ± 10	0.792
Systolic blood pressure, mmHg	137 ± 21	130 ± 30	0.537
Cardiac output, l/min	–	5.01 ± 3.32	–
Cardiac index, l/min/m^2^	–	2.98 ± 1.64	–
Right atrial pressure, mmHg	–	6 ± 6	–
Mean pulmonary artery pressure, mmHg	–	38 ± 8	–
Pulmonary capillary wedge pressure, mmHg	–	12 ± 1	–
Systemic vascular resistance, dyne s/cm^5^	–	1,948 ± 750	–
Total pulmonary vascular resistance, dyne s/cm^5^	–	588 ± 228	–
Pulmonary systemic flow ratio	–	0.98 ± 0.12	–
Pulmonary and systemic resistance ratio	–	0.33 ± 0.11	–

** *Cohort is a subset from a previous analysis (Finsberg et al., 2019). LV, left ventricle; LVEDV, left ventricle end-diastolic volume; LVESV, left ventricle end-systolic volume; LVSV, left ventricle stroke volume; RV, right ventricle; LVEDV, right ventricle end-diastolic volume; LVESV, right ventricle end-systolic volume; LVSV, right ventricle stroke volume*.

### Estimation of Relative Area Change

RAC is defined as *RAC* = (*A*_max_ − *A*_min_)/*A*_min_ (Bellofiore et al., 2013), where *A*_max_ and *A*_min_ are, respectively, the maximum and minimum cross-sectional areas of the MPA were computed for each subject and used to calibrate our computational framework. Measurements of PA areas cross section were performed at a plane normal to the artery. This metric of arterial compliance has been related to the progression of PAH and was associated with the mortality of PAH patients (Gan et al., 2007). The MPA diameter was calculated from the cross-sectional area at the proximal MPA in each patient.

### Reconstruction of 3D Patient-Specific Pulmonary Vasculature

Arteries up to the first three or four generations of the pulmonary arterial tree were reconstructed from the MR images corresponding to the end-diastolic time-point for all subjects (PAH and controls) using a protocol described in details in our previous study (Zambrano et al., 2018). Briefly, an initial geometrical reconstruction of the PA vasculature was obtained using MeVisLab (MeVisLab; Bremen, Germany). These geometries were then imported into the CRIMSON software (CardiovasculaR Integrated Modeling and SimulatiON; www.crimson.software; Xiao et al., 2013; Khlebnikov and Figueroa, 2015) where the final pulmonary geometries were refined and the finite element (FE) meshes were generated. A tetrahedral anisotropic FE mesh with a characteristic element length of 0.7 mm at the center and 0.3 mm at the first five elements adjacent to the arterial wall was generated on each of the refined geometry. The mesh size was selected based on a previous sensitivity analysis (Zambrano et al., 2018).

### Image-Based Fluid Structure Interaction Simulations

Hemodynamic computer simulations were performed on the patient-specific FE pulmonary vasculature models using the CRIMSON software. For these simulations, blood was assumed to behave as a Newtonian fluid with a viscosity 0.04 Pa and the arterial wall was assumed to behave as a linear isotropic elastic membrane with a Poisson ratio ν of 0.5, and a non-uniform wall thickness *h* estimated using a previously reported diameter–thickness relationship (Li et al., 2012) for human PA arteries.

Model parameters were calibrated with patient-specific data using a protocol that was previously described (Zambrano et al., 2018). Briefly, patient-specific volumetric flow rate waveform mapped as a blunt velocity profile and three-element Windkessel parameters ($R_{d}^{i},R_{\text{p}}^{i},C^{i}$) were imposed as inlet and outlet boundary conditions, respectively. On each outlet boundary (*i*), proximal resistances ($R_{\text{p}}^{i}$) were assumed to be equal to the characteristic PA impedance (*R*_p_; **Equation 1a**) and distal resistances ($R_{d}^{i}$) were calculated by subtracting $R_{\text{p}}^{i}$ from a total resistance ($R_{\text{t}}^{i\;}$; **Equation 2**):

(1a, b) $$R_{\text{p}}^{i}=R_{\text{p}}=\frac{\rho C_{\text{ed}}}{A_{\text{MPA}}({\text{t}}_{\text{ed}})};C_{\text{ed}}^{2}=\frac{\frac{2}{3}\sqrt{\pi }Eh}{\rho \sqrt{{\text{A}}_{\text{MPA}}({\text{t}}_{\text{ed}})}},\;$$

(2) $$R_{\text{d}}^{i}=R_{\text{t}}^{i}-R_{\text{p}},\;$$

where ρ, *C*_*ed*_, *A*_MPA_(*t*_ed_), and *h* denote the fluid density, pulse wave propagation speed, MPA cross sectional area at the end-diastolic, and main PA thickness, respectively.

$R_{\text{T}}^{i}$ and *C*^*i*^ for each outlet *i* were then derived from an equivalent total arterial resistance (*R*_T_) and compliance (*C*_T_) by assuming an equal flow split between left and right PA and according their equivalent outlet area (**Equations 3, 4**).

(3a, b) $$R_{\text{T}}^{\text{LPA}}=R_{\text{T}}^{\text{RPA}}=2R_{\text{T}};\frac{1}{R_{\text{t}}^{i}}=\left(\frac{A^{i}}{A^{\text{T}}}\right)\left(\frac{1}{2R_{\text{T}}}\right),\;$$

(4a, b) $$C_{\text{T}}^{\text{LPA}}=C_{\text{T}}^{\text{RPA}}=\left(\frac{C_{\text{T}}}{2}\right);C^{i}=\left(\frac{C_{\text{T}}}{2}\right)\left(\frac{A^{i}}{A^{\text{T}}}\right).\;$$

Initial estimated values of total arterial resistance (*R*_T_) and compliance (*C*_T_) for each subject (**Equation 5**) were iteratively adjusted along with the elastic modulus (*E*) in two nested loops until measured RAC, pulse ( Δ*P* = *P*_sys_ − *P*_dias_), and diastolic (*P*_dias_) pressures were achieved.

(5a, b) $$R_{T}=\frac{{\bar{P}}_{\text{MPA}}}{{\bar{\dot{Q}}}_{\text{MPA}}};C_{T}=\frac{{\dot{Q}}_{\max}-{\dot{Q}}_{\min}}{P_{\text{sys}}-P_{\text{dias}}}\Delta t.\;$$

Here, ${\bar{\overset{∙}{Q}}}_{\text{MPA}},\;{\overset{∙}{Q}}_{\max}$, ${\overset{∙}{Q}}_{\min}$, ${\bar{P}}_{\text{MPA}}$, *P*_sys_, *P*_dias_, and Δ*t* represent MPA mean; maximum and minimum volumetric flow rates; mean; systolic and diastolic pressures; and the time interval between maximum and minimum volumetric flow rates, respectively.

Once calibrated, *E*, *R*_*t*_, and *C*_*t*_ were recorded and time-averaged wall shear stress (TAWSS) was computed from all subjects (PAH and controls).

### Statistical Analysis

Median and interquartile range (IQR) of the calibrated model parameters (*E*, *R*_t_, and *C*_t_), TAWSS, as well as geometrical quantities (*RAC* and diameter) were estimated and used to perform a non-parametric Mann–Whitney *U*-test. Also, Spearman's rank correlation tests were performed to quantify the relationships between hemodynamic quantities and the calibrated model parameters in the PAH (*n* = 6) and the control (*n* = 5) groups. All statistical analyses were performed using the MATLAB software (MathWorks, Natick, Massachusetts, USA) for which a statistical significance of *P* ≤ 0.050 was defined.

## Results

### Arterial Wall Mechanics

**Figure 1:**
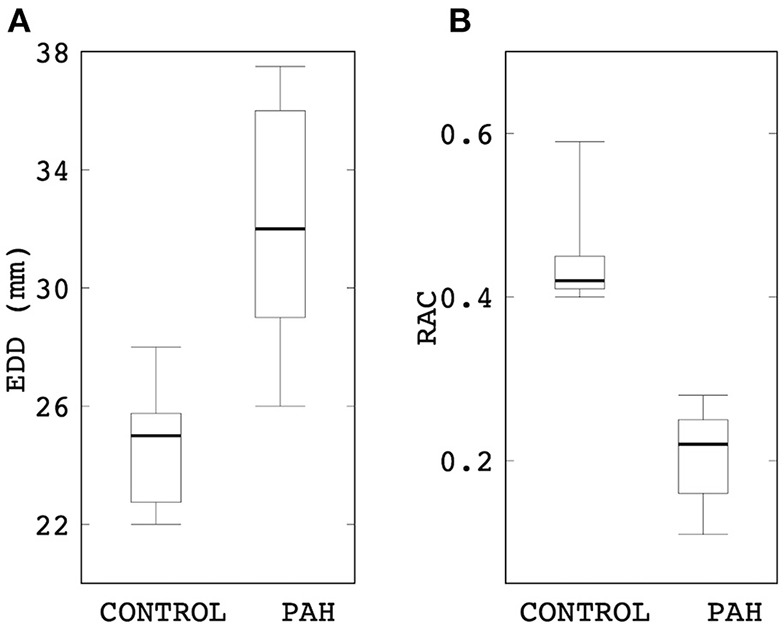
Comparison of **(A)** end-diastolic diameter and **(B)** RAC between control and PAH groups. The PAH group has significant larger diameter (median: 32 mm, IQR: 5.25 mm vs. median: 25 mm, IQR: 2 mm; *P* = 0.015) and lower RAC (median: 0.22, IQR: 0.10 vs. median: 0.42, IQR: 0.04; *P* = 0.004) compared to the control group.

End-diastolic diameter of the MPA in the PAH group was found to be significantly larger (by 28%) than that in the control group (median: 32 mm, IQR: 5.25 mm vs. median: 25 mm, IQR: 2 mm; *P* = 0.015; Figure 1A). On the other hand, *RAC* was also significantly reduced (by about 50%) in the PAH group compared to that in the control group (median: 0.22, IQR: 0.10 vs. median: 0.42, IQR: 0.04; *P* = 0.004; Figure 1B).

**Figure 2:**
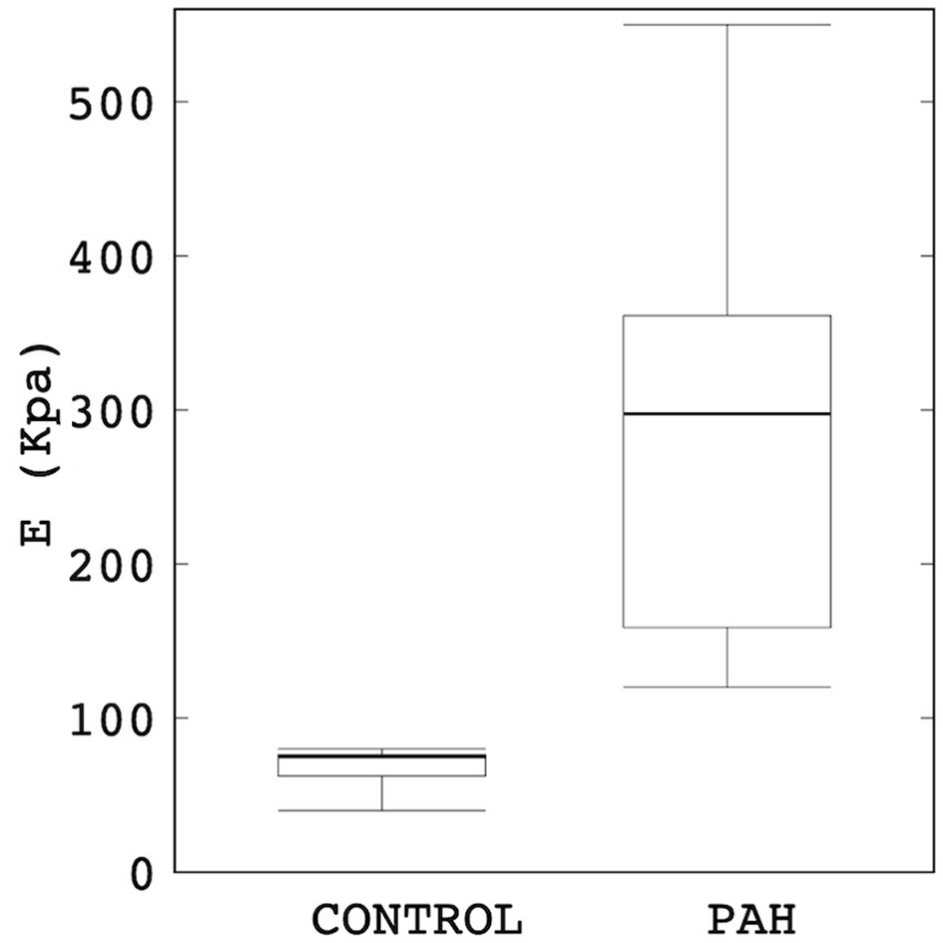
Comparison of the estimated linearized stiffness *E* between PAH and control groups (median: 297 kPa, IQR: 202 kPa vs. median: 75 kPa, IQR: 5 kPa; *P* = 0.007). The PAH group's *E* is approximately four times higher than the control group.

Linearized PA stiffness *E* was found to be significantly higher (by about four times) in the PAH group compared to the control group (median: 297 kPa, IQR: 202 kPa vs. median: 75 kPa, IQR: 5 kPa; *P* = 0.007; Figure 2). Interquartile range of *E* was also substantially smaller in the control group than the PAH group (Figure 2).

**Figure 3:**
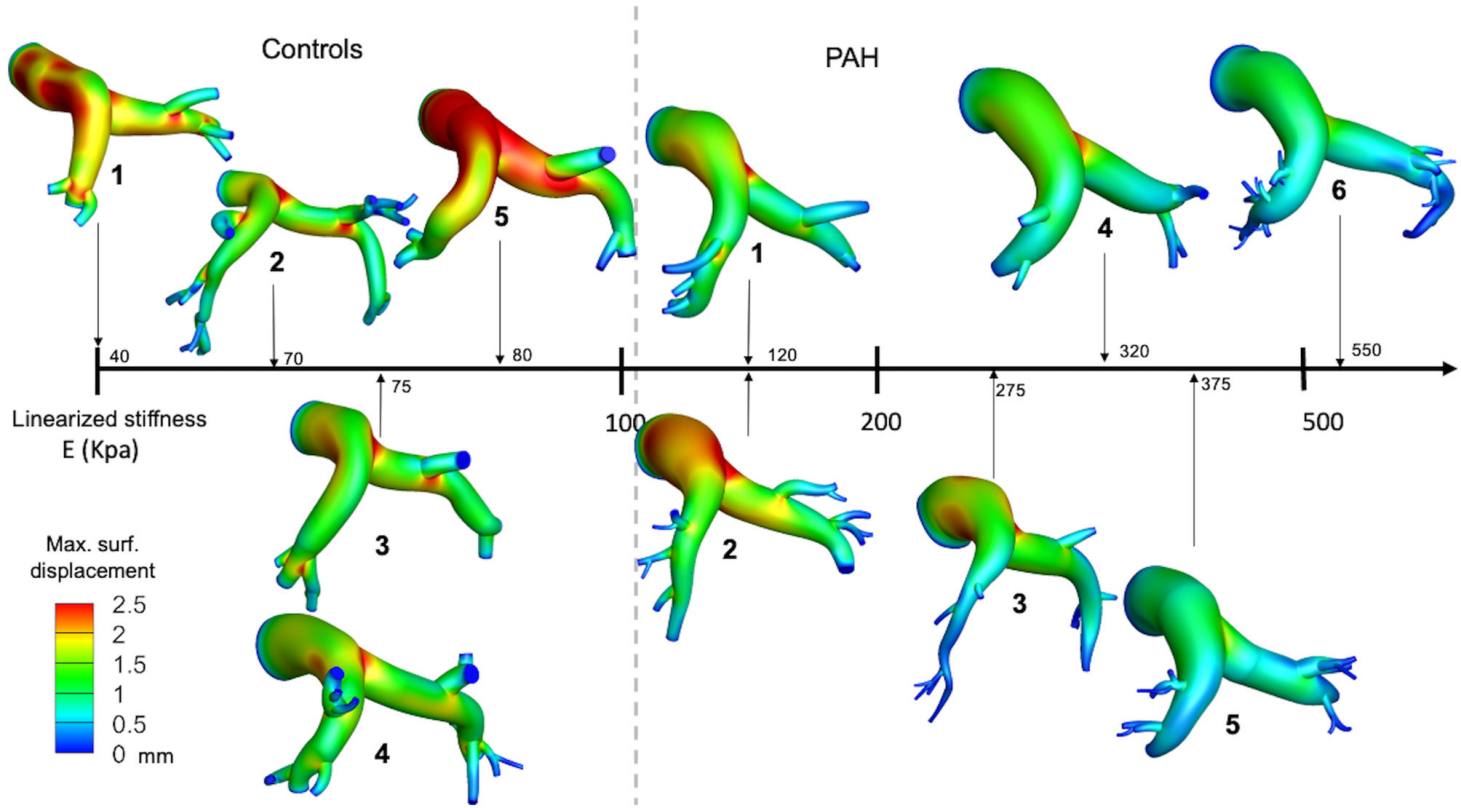
Spatial distribution of the maximum surface displacement in all PAH and control patients organized from the case with the lowest (left) to the highest (right) elastic modulus.

Maximum out-normal surface displacement (over a cardiac cycle) computed using the calibrated model parameters was lower in the PAH group in comparison to the control group. Maximum surface displacement (over the cardiac cycle) was generally found to decrease with increasing linearized stiffness *E* in the PAH patients (Figure 3). Additionally, a larger decrease in maximum surface displacement was found at the distal RPA and LPA than at the proximal MPA regions in the PAH patients with large *E* (i.e., PAH-5, PAH-6; Figure 3). Values of *E* for each patient are listed in Supplementary Table 1 in the supplemetary information.

### Hemodynamics

**Figure 4:**
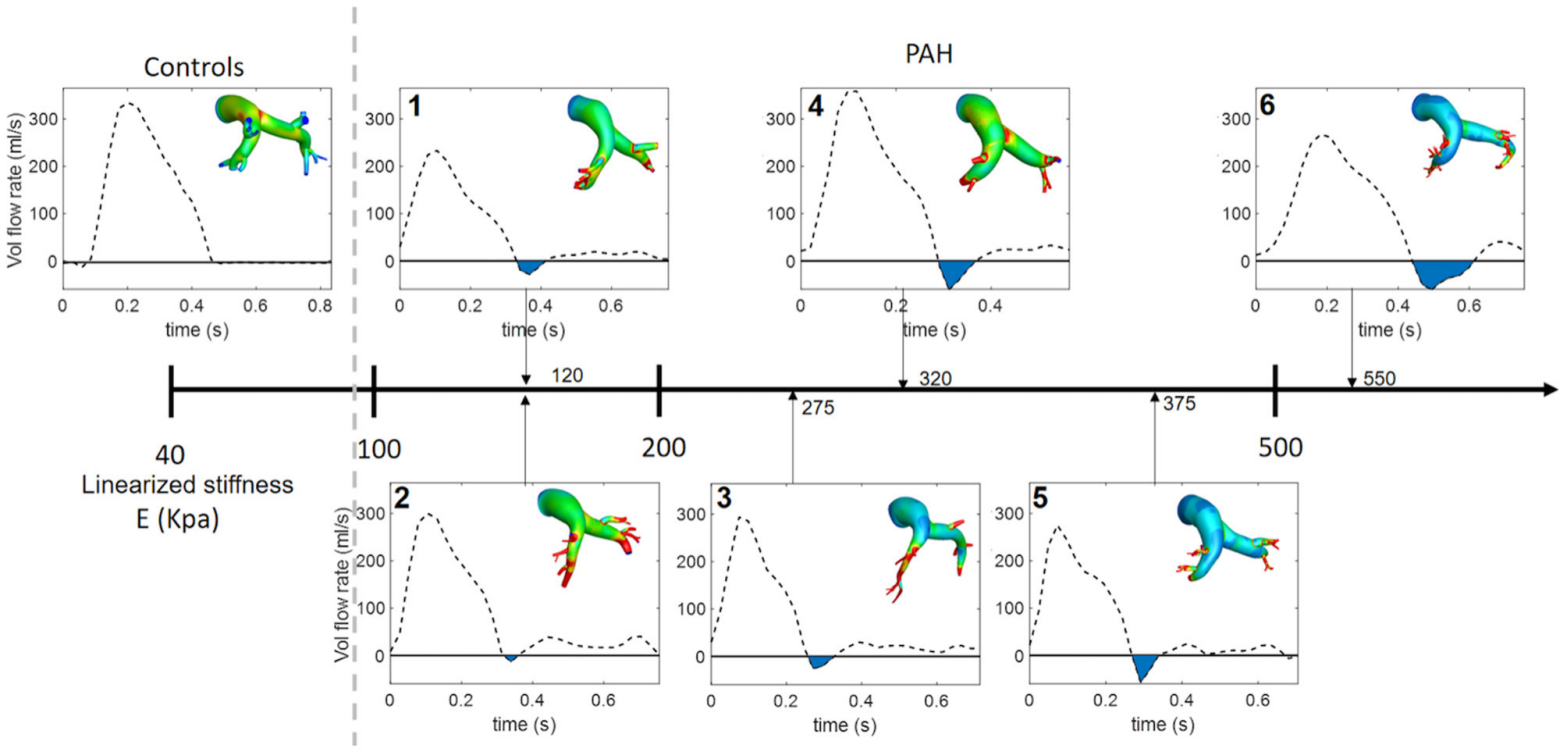
Volumetric flow waveform of control subject CTL-2 as an example of the characteristic wavefrom in controls (left) and measured waveforms for each PAH patient arranged with increasing linearized stiffness *E*. Blue shaded region denotes total regurgitant flow in the proximal PA. The color countour, which is used in Figure 3. represents the maximun surface displacement.

**Figure 5:**
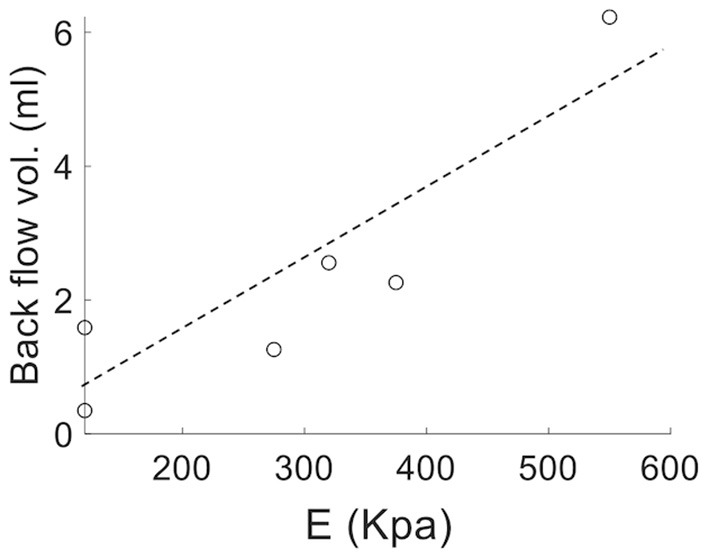
Volume of the total regurgitant flow over a cardiac cycle vs. linearized stiffness *E* for all PAH patients showing a linear correlation (*r* = 0.84, *P* = 0.018).

Analysis of the volumetric flow rate waveforms (measured at the proximal PA using PC-MRI) showed a regurgitant (back) flow after the systolic phase in all PAH patients (Figure 4; blue region). The amount of regurgitant flow was found to increase with increasing linearized stiffness *E*. A strong linear correlation (*r* = 0.84, *P* = 0.018; Figure 5) was found between the total amount of regurgitant flow in the MPA over a cardiac cycle (area of the blue region in Figure 4) and the model estimated linearized elastic modulus *E*.

**Figure 6:**
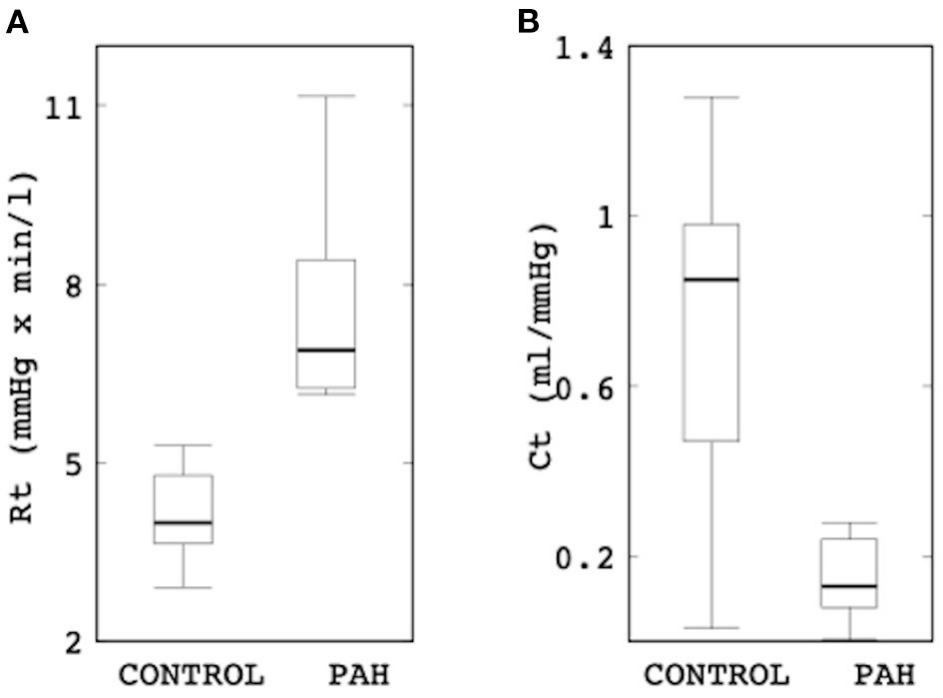
Comparison of **(A)** the total distal resistance (control: median: 3.99 mmHg × min/l, IQR: 1.15 mmHg × min/l vs. PAH: median: 6.89 mmHg × min/l, IQR: 2.16 mmHg × min/l; *P* = 0.002) and **(B)** total distal compliance (control: median: 0.85 ml/mmHg, IQR: 0.51 ml/mmHg vs. PAH: median: 0.13 ml/mmHg, IQR: 0.15 ml/mmHg; *P* = 0.041) between the control and PAH groups.

Comparison of the Windkessel parameters related to the distal vasculature showed that the PAH group has significantly higher distal resistance (median: 6.89 mmHg × min/l, IQR: 2.16 mmHg × min/l vs. median: 3.99 mmHg × min/l, IQR: 1.15 mmHg × min/l; *P* = 0.002; Figure 6) and lower distal compliance (median: 0.13 ml/mmHg, IQR: 0.15 ml/mmHg vs. median: 0.85 ml/mmHg, IQR: 0.51 ml/mmHg; *P* = 0.041; Figure 6) compared to the control group.

**Figure 7:**
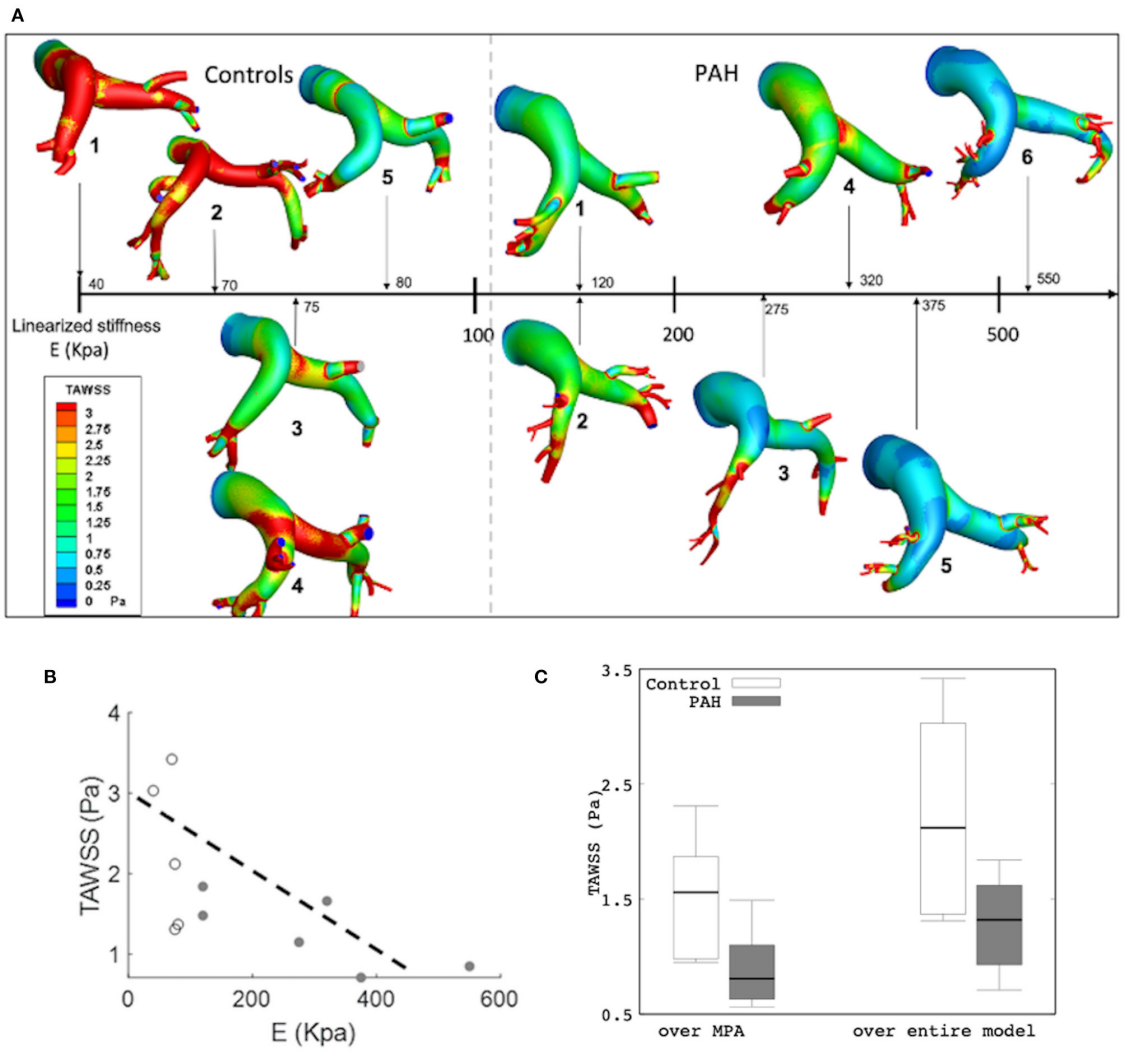
**(A)** Color-coded spatial distribution of TAWSS for all subjects (control and PAH) arranged with increasing linearized stiffness *E*. **(B)** Averaged TAWSS (over the entire model) as a function of *E* showing a weaker correlation (*r* = −0.646; *P* = 0.032) and a stronger correlation (*r* = −0.746; *R* = 0.088) in all and only PAH subjects, respectively. Control (white) and PAH (gray). **(C)** Comparison of TAWSS averaged over the MPA (control: median: 1.56 Pa, IQR: 0.89 Pa vs. PAH: median: 0.81 Pa, IQR: 0.47 Pa) and over the entire model (median: 1.32 Pa, IQR: 0.69 Pa vs. median: 2.12 Pa, IQR: 1.66 P; *P* = 0.062) between PAH and control groups.

Results from the simulation also showed differences in the spatial distribution of TAWSS in all patients. On average, mean TAWSS was higher in the control group than the PAH group (Figure 7A). Similar to the relationship between maximum surface displacement and linearized elastic modulus *E*, TAWSS decreased with increasing *E* (Figure 7A). Specifically, TAWSS averaged over the entire PA and MPA segments was lower in the PAH than the control group (entire PA median: 1.32 Pa, IQR: 0.69 Pa vs. median: 2.12 Pa, IQR: 1.66 Pa; *P* = 0.062; MPA segment median: 0.81 Pa, IQR: 0.47 Pa vs. median: 1.56 Pa, IQR: 0.89 Pa; *P* = 0.026; Figure 7B). Mean TAWSS over the entire PA and over MPA showed strong correlations with the linearized stiffness *E* when all subjects (PAH + control) were considered (entire PAs: *r* = −0.76; *P* = 0.038; Figure 7C; MPAs: *r* = −0.79; *P* = 0.029). When only PAH patients were considered, however, TAWSS averaged over the entire PA was strongly correlated with *E* (*r* = −0.72; *P* = 0.051; Figure 7C). Geometric, hemodynamics, and wall mechanical quantities for each control and PAH subject are summarized in Supplementary Table 1 in supplemetary information.

## Discussion

We have applied a patient-specific computational framework to investigate the interactions between vessel wall mechanics and hemodynamics in the PA of six PAH patients and five control subjects. The key findings are as follows: (1) the linearized elastic modulus *E* of the PA is significantly elevated in PAH patients compared to the control subjects, (2) *E* is directly correlated with the total regurgitant flow in the PA, (3) TAWSS is inversely associated with the increase in the linearized elastic modulus *E* in PAH patients, and (4) distal resistances *R*_*t*_ and compliance *C*_*t*_ are, respectively, higher and lower with PAH.

Pulmonary arterial stiffness is recognized as a key marker for early detection of the PAH (Sun and Chan, 2018). It was shown that an increase in PA stiffness occurs much earlier during the development of PH (regardless of the pathology; Sanz et al., 2009). Clinical studies have reported a stiffer (Ray et al., 2019) and an enlarged (Lange et al., 2013) proximal PA artery associated with PAH and have suggested that the changes in PA diameter and/or RAC can be used to characterize changes in PA stiffness, which in turn can serve to depict the progression of the disease. The reliability of these metrics in reflecting arterial stiffening is, however, questioned by others who suggest that the relationship of these parameters may be affected by other diseases (e.g., parenchymal lung disease; Tan et al., 1998; Raymond et al., 2014). Findings of a larger PA diameter (Figure 1A) and a lower RAC (Figure 1B) in the PAH patients compared to control subjects shown here are consistent with clinical studies (Swift et al., 2017; Tonelli et al., 2017). Our analysis also went beyond these purely geometrical and kinematics metrics, showing that the linearized stiffness *E* of the proximal PA is approximately four times stiffer in the PAH group (Figure 2B) than the control group. While mechanobiological changes in the PA are still unknown (Hunter et al., 2011), arterial stiffening of systemic arteries due to hypertension was suggested to be associated to the increase in extracellular matrix (ECM) components such as elastic and collagen fibers (Diez, 2007). Animal PAH models have also observed these ECM changes in PA arteries (Meyrick and Reid, 1979, 1980; Poiani et al., 1990; Kobs et al., 2005), which were associated to an increase in PA wall thickness (Dieffenbach et al., 2018). Correspondingly, our study suggests that the linearized stiffness (estimated at the proximal arteries) may help to characterize the changes that are useful in depicting the clinical evolution of the disease. Furthermore, its relationship with other hemodynamic parameters such as volumetric flow profiles (Figures 4, 5) and TAWSS (Figure 6) can potentially be used to reflect these changes.

Low and reverse flow profiles in the MPAs have been observed between early systolic and end-systolic phases in PAH patients (Murata et al., 2000; Helderman et al., 2011; Hu et al., 2012; Odagiri et al., 2016). Specifically, imaging studies have reported retrograded flow in the PA and the formation of vortical structures in the enlarged proximal arteries in PAH patients that are both associated with RV dysfunction (Helderman et al., 2011). These features were also associated with a systolic deceleration in the flow profile at the RV outflow tract of PAH patients and with a poor prognosis (Arkles et al., 2011). Here, reverse flow is also present at the end of the systolic phase in PAH patients (Figure 4). Interestingly, the total amount of reverse flow (in a cardiac cycle) is found to be strongly correlated to the linearized stiffness (Figure 5), suggesting that the amount of PA regurgitant flow (that can be measured non-invasively) may be used as a metric for characterizing changes in the PA intrinsic stiffness.

Another hemodynamic parameter recently suggested as potential marker of PAH is the time-averaged WSS (TAWSS). A comparison of the estimated TAWSS values between PAH and control groups shows an interesting trend. Besides finding that TAWSS is lower at the proximal PA arteries in PAH patients than the control subjects (Figure 7C); which is consistent with a previous analysis (Tang et al., 2012), we also found that TAWSS is inversely correlated to the linearized stiffness (Figure 7C). Although the correlation between TAWSS and *E* was not reached for the significance (*P* = 0.051) since our threshold was set to 0.050, given the small number of cases, the correlation in six PAH patients can be indicative of a potential for the significant correlation if tested in a larger cohort. Arguably, this borderline significant results could be attributed to differences in using nominal values such as volumetric inflow and PA diameters to determine WSS (Odagiri et al., 2016). An imaging analysis has found a significant decrease in WSS at the proximal PA of adolescents and children PAH patients under preserved flow rates (Truong et al., 2013). These low levels of TAWSS were suggested to be linked to abnormal and regurgitant PA back fluid profiles (Odagiri et al., 2016) and the increase in vascular resistance (Schäfer et al., 2016; Zambrano et al., 2018). Also, the decrease in TAWSS levels has been associated with an increase in PA stiffness (Friesen et al., 2019), which agrees with our observations. Experimental studies have shown that WSS influences endothelial cell function that triggers a biomechanical arterial response that promotes an arterial stiffening (Ben Driss et al., 2000).

There are some limitations associated with this study, some which are already summarized in a previous study (Zambrano et al., 2018). Briefly, the coupled momentum formulation assumes the vessel wall to behave as a linear elastic material, which is a first-order approximation of the vessel wall mechanics. Model segmentation of the distal vasculature is subjected to a larger variability in some cases due to clinical MR image resolution limitation. Flow and pressure data for control subjects are not available. We have mitigated this limitation, however, by scaling the flow waveform so that the total outflow at the MPA is the same as the RV stroke volume measured from the MR image. Additionally, statistical comparison and correlations performed here provide some insights about differences and potential associations of the parameters among all patients. However, a higher number of patients should be analyzed before reaching stronger conclusions.

## Conclusion

Pulmonary arterial stiffness and hemodynamic metrics measured at the proximal PA are believed to be potential biomechanical markers for the identification of PAH disease. Our study, using a novel FSI computational framework and clinical patient data, analyzed these aspects and their relationship in six PAH patients and five control subjects. Our results showed that proximal PA stiffness is associated to PA hemodynamic parameters such as TAWSS and back regurgitant flow. Although study with a larger cohort of patients would need to be conducted to reach stronger conclusions, these associations highlight the potential of hemodynamic parameters of becoming important biomarkers to stratify PAH severity in terms of PA stiffness.

## Data Availability Statement

The original contributions presented in the study are included in the article/Supplementary Material, further inquiries can be directed to the corresponding author/s.

## Ethics Statement

The study was approved by the SingHealth Centralised Institutional Review Board and written informed consent was obtained from all participants.

## Author Contributions

LL, LZ, and SB designated and directed the overall project. BZ and NM implemented all the analysis and were involved in the evaluation of the results and preparation of the manuscript. XZ, J-LT, and LZ contributed to patient and image acquisition. BZ, XZ, J-LT, and LZ contributed to data analysis and statistics. CF contributed to model development and model parameter estimation. All the authors contributed to the evaluation of the results, discussion, and the overall manuscript.

## Conflict of Interest

The authors declare that the research was conducted in the absence of any commercial or financial relationships that could be construed as a potential conflict of interest.
