# Supplementary material for: Patient-Specific Computational Analysis of Hemodynamics and Wall Mechanics and Their Interactions in Pulmonary Arterial Hypertension
Source: Front Bioeng Biotechnol. 2021 Jan 28;8:611149. doi: 10.3389/fbioe.2020.611149 (PMC7901991; doi:10.3389/fbioe.2020.611149)
Supplement: Supplementary file 1 [file Table_1.pdf]

TABLE S1. Calculated geometric, hemodynamic and wall parameters for control (CTL) and PAH patients.

CONTROLS

#	MPA TAWSS (Pa)	Prox. PA TAWSS (Pa)	Elastic Modulus E (Kpa)	MPA RAC	MPA ED D (mm)	Rt (mmHg x min/l)	Ct (ml/mmHg)	MPA Back Flow
1	1.87	3.03	40	0.59	22.0	3.99	0.467	-
2	2.31	3.42	70	0.41	23.0	2.91	0.850	-
3	1.56	2.12	75	0.45	28.0	3.64	1.280	-
4	0.98	1.31	75	0.40	25.0	5.29	0.030	-
5	0.95	1.37	80	0.42	25.0	4.79	0.980	-

PAH

#	MPA TAWSS (Pa)	Prox. PA TAWSS (Pa)	Elastic Modulus E (Kpa)	MPA RAC	MPA ED D (mm)	Rt (mmHg x min/l)	Ct (ml/mmHg)	MPA Back Flow (ml)
1	0.93	1.48	120	0.23	26.0	6.16	0.158	1.59
2	1.15	1.84	120	0.28	29.0	6.50	0.004	0.35
3	0.69	1.15	275	0.26	36.0	8.79	0.096	1.26
4	1.49	1.66	320	0.21	31.0	6.14	0.077	2.56
5	0.56	0.71	375	0.14	33.0	11.15	0.262	2.26
6	0.61	0.85	550	0.11	37.5	7.28	0.280	6.23

MPA=Main pulmonary artery, Prox. PA= proximal pulmonary arteries, RAC=relative area change, EDD= end diastolic diameter, Rt=total distal resistance, Ct= total distal compliance,
